# A new shape for an old function: lasting effect of a physiologic surgical restoration of the left ventricle

**DOI:** 10.1186/1749-8090-1-40

**Published:** 2006-11-03

**Authors:** Marco Cirillo, Andrea Amaducci, Emmanuel Villa, Margherita Dalla Tomba, Federico Brunelli, Zen Mhagna, Giovanni Troise, Eugenio Quaini

**Affiliations:** 1 Department of Cardiovascular Surgery, Cardiac Surgery Unit, Poliambulanza Foundation Hospital, Brescia, Italy; 2 Department of Cardiovascular Surgery, Cardiology Unit, Echocardiography Laboratory, Poliambulanza Foundation Hospital, Brescia, Italy; 3 University of Milan, Milan, Italy

## Abstract

**Background:**

Long-term morphofunctional outcome may vary widely in surgical anterior left ventricular wall restoration, suggesting variability in post-surgical remodeling similar to that observed following acute myocardial infarction. The aim of this pilot study was to demonstrate that surgical restoration obtained with a particular shape of endoventricular patch leads to steady morphofunctional ventricular improvement when geometry, volume and residual akinesia can be restored as normal as possible.

**Methods:**

This study involved 12 consecutive patients with previous anterior myocardial infarction, dilated cardiomyopathy and no mitral procedures, who underwent left ventricular reconstruction and coronary revascularization between May 2002 and May 2003 using a small, narrow, oval patch aiming at a volume ≤ 45 mL/m^2 ^with elliptical shape. Eleven geometric parameters were examined preoperatively and at least 3, 12 and 24 months after the operation by serial echocardiographic studies and evaluated by paired *t *test taking the time of surgery as a starting point for remodeling.

**Results:**

All patients were in NYHA class 1 at follow-up. Patch geometry obtained a conical shape of the ventricle with new apex, physiologic rearrangement of functioning myocardial wall and small residual akinesia. Ventricular changes at the four time-points showed that all parameters improved significantly compared to preoperative values (end-diastolic volume = 184.2 ± 23.9 vs 139.9 ± 22.0, p = 0.001; vs 151.0 ± 33.8, p = 0.06; vs 144.9 ± 34.0, p = 0.38; end-systolic volume = 125.7 ± 20.6 vs 75.2 ± 14.1, p = 0.001; vs 82.1 ± 23.9, p = 0,18; vs 77.1 ± 19.4, p = 0.41) without further changes during follow-up except for wall motion score index (2.0 ± 0.2 to 1.7 ± 0.2, to 1.4 ± 0.2, to 1.3 ± 0.2) and percentage of akinesia (30.4 ± 7.5 to 29.3 ± 4.2, to 19.8 ± 11.6, to 14.5 ± 7.2) which slowly and significantly improved suggesting a positive post-surgery remodeling.

**Conclusion:**

Ventricular reconstruction caring of physiological shape, volume, revascularization and residual akinesia obtained a steady geometry. Positive remodeling and equalization of geometrical outcome may persistently prevent long-term redilation.

## Background

**I**schemic disease is the most frequently identified specific cause of dilated cardiomyopathy, accounting for more than 60% of patients with symptomatic heart failure and many more with asymptomatic left ventricular dysfunction. Reconstructive ventricular surgery for ischemic cardiomyopathy has attracted increasing attention over recent years as a result of studies aimed at clarifying the functional structure [1], myocyte biology [2], mechanics and geometry of normal and remodeled left ventricle (LV). Although a number of surgical techniques are still *sub judice *[3-5], current opinions underline the importance of rebuilding a more physiological ventricular chamber. Reported outcomes vary widely [6-9], demonstrating that surgical treatment is the starting point of a complex remodeling process characterized by the uncertainty of long-term LV geometrical changes.

We here describe such changes as measured by eleven echocardiographic parameters over a follow-up period of more than two years in a series of patients treated with endoventricular narrow, oval patch restoration. We then provide an original comparison of the earliest and latest follow-up steps, taking the restored ventricle as a new starting point for remodeling.

## Methods

### Patients

Between May 2002 and May 2003, sixteen consecutive patients with previous anterior myocardial infarction and ischemic cardiomyopathy (end-systolic volume index of >50 mL/m^2^, ejection fraction of ≤ 35%, dominant anterior akinesia/dyskinesia [10,11] and prevalent heart failure symptoms) underwent left ventricular restoration surgery and coronary revascularization. Informed consent was obtained from each participant. The analysis excludes the three who underwent an associated mitral valve procedure, and one who died of non-cardiac causes during the follow-up period, and therefore involved 12 patients who were examined preoperatively, and at least 3, 12 and 24 months after the operation.

### Definitions

All of the clinical definitions were taken from the 1998 data collection form of the Society of Thoracic Surgeons National Cardiac Surgery Database.

Anterior MI: All anterior, anterolateral and anteroapical myocardial infarctions with septal involvement.

Preoperative assessment (Preop): preoperative status.

Follow-up 1 (FU 1): First follow-up at least 3 months after the operation.

Follow-up 2 (FU 2): Second follow-up at least 12 months after the operation.

Follow-up 3 (FU 3): Third follow-up at least 24 months after the operation.

Normalisation of ESVI: Postoperative reduction of end-systolic volume index to ≤ 45 mL/m^2 ^(with a tolerance of +1.5 mL/m^2^); we utilized this variable as a cut-off value to evaluate ventricular redilation at follow-up: left ventricular end-systolic volume index can be used as a prognostic factor insofar as a value of >45 mL/m^2 ^often leads to clinical heart failure [12].

### Echocardiographic measurements

All of the pre- and postoperative echocardiographic studies were performed by the same echocardiographer, who was blinded to the aim of the study, using an Acuson Sequoia 512 (Acuson Corporation, Mountain View, CA) equipped with 2.5–3.5 MHz transducers. The preoperative examinations were performed 0–7 days before surgery; the postoperative echocardiograms used for this study were recorded at least three months (FU 1: mean = 3.3 ± 0.3 months), 12 months (FU 2: mean = 12.2 ± 0.2 months) and 24 months (FU 3: mean = 27.3 ± 2.9 months). Mitral regurgitation (MR) was quantified using a four-point Doppler scale. Eleven LV geometrical and functional parameters were evaluated: end-diastolic (EDD) and end-systolic (ESD) diameters; end-diastolic and end-systolic volumes, including absolute (EDV, ESV) and indexed values (EDVI, ESVI); diastolic (LLD) and systolic (LLS) longitudinal length; ejection fraction (EF); wall motion score index (WMSI) and percentage of akinesia (AKIN). These parameters were defined according to the Recommendation of the American Society of Echocardiography [13].

### Surgical details

All of the operations were performed by means of a median sternotomy using a mild hypothermic (33°C) cardiopulmonary bypass, aortic cross-clamping, intermittent cold blood cardioplegia and warm reperfusate.

**Figure 1:**
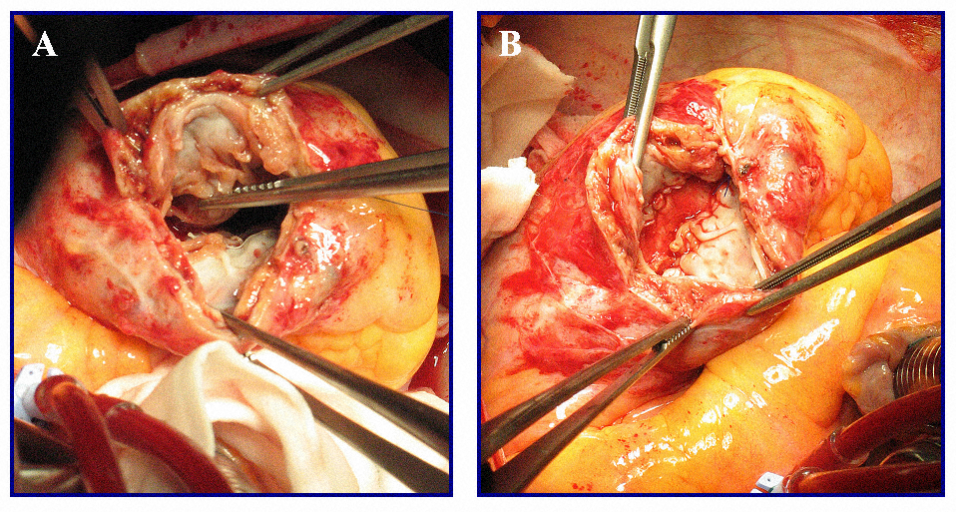
**Surgical view through left ventriculotomy**. A: Border zone between normal and necrotic myocardium; the forceps shows the effect of bringing the lateral wall towards the posterior septum along a linear direction. B: The narrow oval-ended patch rebuilds the apex, brings the lateral functioning wall near to the posterior part of the septum along a physiologic rearrangement and leaves a small akinetic part.

**Figure 2:**
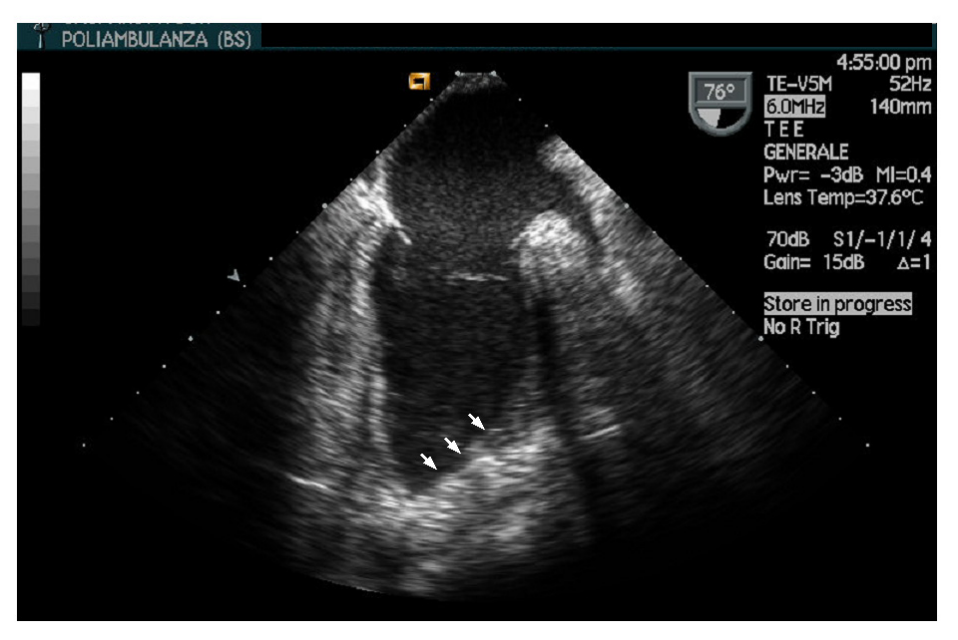
**Postoperative transesophageal echocardiographic long axis view of a restored left ventricle**. A conical left ventricle is restored. Arrows show the position of the patch. The new apex is evident.

Left ventriculotomy was performed laterally and parallel to the course of the left anterior descending artery. The LV restoration surgical technique was endoventricular plasty using a small, narrow, oval patch (minor axis 1.8 ± 0.3 cm; major axis 3.8 ± 0.5 cm; mean area = 5.6 ± 1.7 cm^2^) obliquely positioned inside the ventricle with one end high in the interventricular septum and the other rebuilding a ventricular apex; the major axis was proportional to the length (the extension) of necrotic area. The new apex is localized at the end of necrotic tissue of the old apex (usually more postero-lateral than the anatomic one) and at the joining point of the two suture lines (septal and lateral) along the patch. An autologous, glutaraldehyde-treated pericardial patch was used in all cases. Fontan purse-string was not used to avoid circularity of the final defect and distortion of myocardial fibers bands: in this way the patch approaches the left lateral wall to the posterior interventricular septum along a straight line in a more physiologic arrangement (Figure 1). The residual cavity has a conical shape (Figure 2). In all cases, the ventriculotomy was closed by overlapping the free edges in a "vest-over-pants"-type closure in order to occlude the excluded chamber. All of the patients received coronary artery bypass grafts in order to ensure complete revascularization. The left anterior descending artery was revascularized in all cases.

### Statistical analysis

The clinical, echocardiographic, operative and outcome data were prospectively collected in our Institutional database, and statistically analysed using SPSS 11.0 software (SPSS Inc., Chicago, IL, USA). The continuous variables are expressed as mean values ± SD. The ordinal variables were cross-tabulated using Pearson's χ-squared test. The statistical analyses were used to describe two scenarios:

1. Repeated Measures ANOVA compared four time-points in an analysis of variance with time of measurement (Preop vs. FU 1 vs. FU 2 vs. FU 3) as a within-subjects factor; the sphericity assumption was not met only for ESD and the Huynh-Feldt correction was applied. Post-hoc comparisons were made using the Bonferroni's adjustment for multiple comparisons. The data were plotted as mean values ± 2 standard error of the mean.

2. Given the substantial geometrical modifications obtained by surgery, and the intention to restore nearly normal geometry, we then considered FU 1 status as a new starting point for ventricular remodeling and used paired *t *test to compare FU 1 *vs *FU 3 (considered as a 24-months follow-up control of the "new" ventricles) in a within-subject modeling.

A *P *value of ≤ 0.05 was considered as significant.

## Results

The patients' clinical data are summarised in Table 1. None of the patients was rehospitalised or experienced the recurrence of preoperative symptoms during the follow-up period. All of the patients received postoperative maximal medical therapy and were in NYHA class 1 at all follow-up times.

**Table 1:** Clinical characteristics

	*No. 12*
Age (years)	66.0 ± 6.4 (52–75)
Gender (M/F)	12/0
Body surface area (m^2^)	1.78 ± 0.1 (1.57–1.95)
Diabetes	4
Hypertension	6
Elective/Urgent	11/1
Euroscore	6.1 ± 1.8
N.Y.H.A. class	2.58 ± 0.9
Time interval from MI (months)	189.1 ± 112.1 (4.5–346.3)
Mono/bi/three-vessels disease	1/6/5
Mean grafts/patient	2.5 ± 0.9

The patient who died 13.3 months after the operation was an 81-years-old male with non-cardiac worsening of pre-existing renal and respiratory failure. His basal echocardiogram showed EDV = 220 mL, ESV = 146 mL, EF = 33%, WMSI = 2,31 and grade 2 MR. His last echocardiographic examination after 7.23 months showed EDD = 49 mm; ESD = 39 mm; ESV = 55 mL, EDV = 144 mL, ESVI = 30.5 mL/m^2^, EDVI = 80 mL/m^2^, EF = 60%, WMSI = 1.7, and a grade 1 mitral regurgitation.

### First scenario: time-course of geometric ventricular changes (repeated measures analysis)

**Figure 3:**
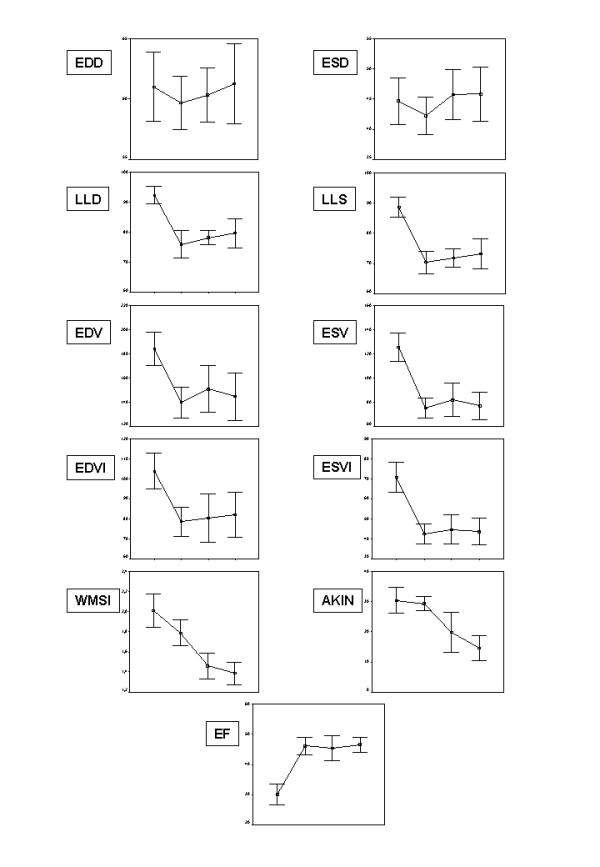
**Graphical plots of selected variables at preoperative, FU 1, FU 2 and FU 3 steps**. Labels link the corresponding data in Table 2. Plots describe mean ± 2 standard error of the mean (SEM). Abbreviations as in 'Echocardiographic measurements'.

Numerical data are reported in Table 2 and corresponding graphical plots in Figure 3. All geometrical data significantly improved at FU 1 *vs *Preop but EDD (*P *= 0.27) and percentage of akinesia (*P *= 0.54). Between FU 2 *vs *FU 1, only WMSI (*P *= 0.002) and percentage of akinesia (*P *= 0.007) significantly improved, and none of the parameters was significantly different from FU 2 at FU 3. Pairwise comparisons showed: significant differences between Preop and each follow-up time in terms of diastolic and systolic lengths, absolute and indexed volumes, and ejection fraction; no differences in any parameter between the latest two follow-up times; a particular behaviour of WMSI and percentage of akinesia.

**Table 2:** Repeated Measures ANOVA at four time-points and Bonferroni's test for post-hoc comparisons.

*Parameters*	PREOP (n = 12)	FU 1 (n = 12)	FU 2 (n = 12)	FU 3 (n = 12)
**EDD**	61.0 ± 4.9	59.6 ± 3.7	60.3 ± 3.9	61.2 ± 5.7
**ESD**	44.6 ± 6.7	42.2 ± 5.4 *	45.7 ± 7.3	45.8 ± 7.6
**LLD**	92.2 ± 5.0	76.0 ± 8.0 *	78.2 ± 3.9	79.7 ± 8.4
**LLS**	88.6 ± 5.7	70.4 ± 6.5 *	71.8 ± 5.2	73.1 ± 8.4
**EDV**	184.2 ± 23.9	139.9 ± 22.0 *	151.0 ± 33.8	144.9 ± 34.0
**EDVI**	103.8 ± 15.2	78.7 ± 12.7 *	80.5 ± 21.0	82.0 ± 19.5
**ESV**	125.7 ± 20.6	75.2 ± 14.1 *	82.1 ± 23.9	77.1 ± 19.4
**ESVI**	70.8 ± 12.6	42.4 ± 8.6 *	44.7 ± 12.6	43.7 ± 11.3
**WMSI**	2.0 ± 0.2	1.7 ± 0.2 *	1.4 ± 0.2 †	1.3 ± 0.2
**AKIN**	30.4 ± 7.5	29.3 ± 4.2	19.8 ± 11.6 †	14.5 ± 7.2
**EF**	30.0 ± 5.8	46.1 ± 4.9 *	45.4 ± 7.1	46.6 ± 4.2

Labels link the corresponding plots in Figure 3. Values are mean ± SD; * = statistically significant, FU1 vs PREOP; † = statistically significant, FU2 vs FU1. Abbreviations as in 'Echocardiographic measurements'.

All of the WMSI comparisons were significant except between FU 2 and FU 3, with the values decreasing from 2.0 ± 0.2 (Preop) to 1.7 ± 0.2 (FU 1), 1.4 ± 0.2 (FU 2), and 1.3 ± 0.2 (FU 3). The differences in percentage of akinesia between Preop and FU 1, and between FU 2 and FU 3, were not significant, but the other comparisons were highly significant, with the values decreasing from 30.4 ± 7.5 (Preop) to 29.3 ± 4.2 (FU 1), 19.8 ± 11.6 (FU 2, *P *= 0.04 vs FU 1), and 14.5 ± 7.2 (FU 3, *P *< 0.001 vs FU 1).

### Second scenario: remodeling process of restored ventricles (paired t test)

Considering postoperative FU 1 as a new starting point for the remodeling of the restored ventricles, we compared echocardiographic measurements at this time *vs *FU 3, representing a time interval of 23.9 ± 2.8 months. There was a significant correlation between EDV (r^2 ^= 0.69), EDVI (r^2 ^= 0.62), ESV (r^2 ^= 0.56) and ESVI (r^2 ^= 0.49), and paired *t *test showed a highly significant difference only with regards to WMSI and percentage of akinesia (*P *< 0.001) (Table 3).

**Table 3:** P values of paired samples correlations and t test considering FU 1 as baseline and FU 3 as 24-months follow-up.

*Parameters*	Correlations	Paired t tests
End-diastolic diameter	0.55	0.38
End-systolic diameter	0.42	0.17
Longitudinal length – diastolic	0.18	0.26
Longitudinal length – systolic	0.75	0.27
End-diastolic volume	*0.001*	0.40
End-diastolic volume index	*0.002*	0.36
End-systolic volume	*0.005*	0.61
End-systolic volume index	*0.01*	0.57
Wall motion score index	0.51	<*0.001*
Percentage of akinesia	0.17	<*0.001*
Ejection fraction	0.08	0.72

### Mitral regurgitation

Seven patients showed no mitral regurgitation (MR) preoperatively, two showed grade 1, and three grade 2. These patients with MR were not indicated to perform mitral surgery based on geometrical (papillary muscles displacement) or functional (ischemic area) parameters to the thinking that surgical restoration and revascularization could correct it. Mitral regurgitation remained grade 0 at FU 3 in five of the patients in the first group, whereas one developed grade 1, and one grade 2 MR; neither of the Preop grade 1 patients experienced MR during follow-up. All of the patients with Preop grade 2 MR showed no MR at FU 2, but MR had returned to being grade 2 by FU 3. Pearson's χ-squared test showed significance when the presence of MR at FU 3 was tabulated against the normalisation of ESVI at FU 1 (*P *= 0.03) and FU 3 (*P *= 0.005), but not at FU 2 (*P *= 0.38).

### "Normalisation" of ESVI

**Figure 4:**
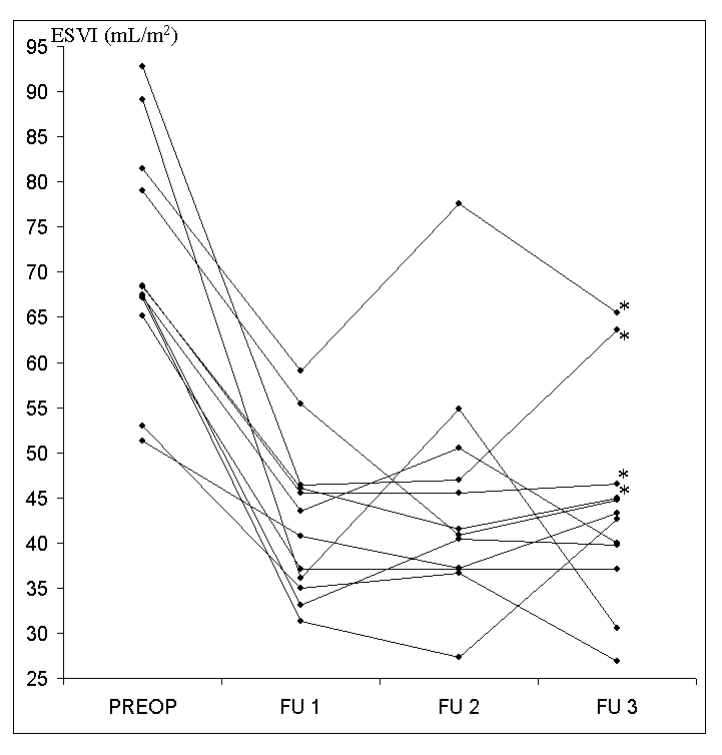
**Time-course of end-systolic volume index for each patient**. Singles values of ESVI and their modifications during time. Note that preoperative larger ventricles have wider changes during time. '*' Flags grade 2 mitral regurgitation.

Ten patients showed normalised ESVI at FU 1 (Figure 4). Two of them lost their normalised value at one-year follow-up (54.89 and 50.55 mL/m^2^) and regained it at FU 3 (30.5 and 40 mL/m^2^). One of them lost his normalised value at FU 3 (63.6 mL/m^2^) and developed grade 2 MR.

Of the 2 patients with a postoperative ESVI of >45 mL/m^2^, one normalised the value at FU 2 (with no MR) and one did not (with grade 2 MR). This means that practically only one patient lost his normalised value over time, and that 1 positively remodeled his ventricle at later follow-up time, related to the absence of MR *or *a volume near the cut-off value. Finally, 10 patients (83.3%) have a normalized ESVI at late follow-up.

## Discussion

The results of left ventricle surgical restoration observed in this homogeneous series of consecutive ischemic cardiomyopathy patients without any preoperative characteristics (posterior infarction, severe mitral regurgitation) or postoperative events (recurrent angina or myocardial infarction, the need for new revascularisation, the onset of severe MR) representing confounding risk factors for unfavourable remodeling, show a steady geometrical outcome.

The repeated measures analysis demonstrated that the substantial morphofunctional changes induced by restoration are maintained over time. The constant improvement in WMSI and percentage of akinesia indicates the slow but continuous positive remodeling of the new ventricles. This may have been due to the recruitment of neighbouring and remote segments of LV wall, which regains its normal physiology as a result of the normalized LV volumes and geometry. This continuous process appears to have a crucial point at about one-year time interval from surgery, suggesting that at least one year is required to stabilize the surgical result and to obtain a steadier geometry. It may also mean that the earlier results of this type of surgery cannot be considered definite, and that long-term repeated observations are necessary to confirm post-surgical LV remodeling. The relevance of the fluctuation during the first postoperative year is also shown by the behaviour of MR and of the "normalization" of ESVI: the greatest differences *vs *FU 1 and FU 3 were observed at FU 2.

### Volume and shape normalization

Surgical restoration replaces the infarct scar with a smaller surgical scar, and tries to modify ventricular geometry in such a way as to recreate a nearly normal ventricle. The structural anatomy of the myocardium cannot be rebuilt, and so it is necessary to give the LV as normal a shape and volume as possible in order to increase the likelihood of it regaining its physiological status [14-16] and to eliminate a trigger for neurohormonal changes [17].

End-systolic volume index normalisation is a good reference value that assures positive remodeling over time. Our findings show that 90% of the ventricles with a surgically normalised ESVI remained unchanged, whereas only 50% of the non-normalised ventricles positively remodeled to a value of ≤ 45 mL/m^2^. We therefore obtained a good and steady geometrical outcome mainly in patients with a normalised ESVI and without mitral regurgitation.

**Figure 5:**
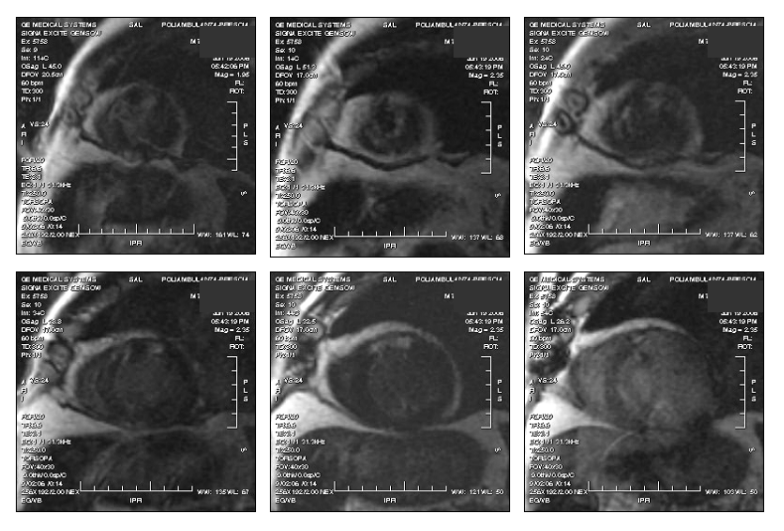
**Magnetic resonance Delay enhancement in short axis views**. The sequence shows completely silent delay enhancement images except for the small area of the patch, which is surrounded with normally functioning myocardium. The patient performed magnetic resonance at latest follow-up visit.

The patch plays a key role in surgical restoring: it replaces the akinetic/dyskinetic myocardium and becomes the starting point of a new remodeling process. In our series, we used a small, narrow, oval, obliquely oriented patch in order to rebuilding the apex, bringing the lateral functioning wall near to the posterior part of the septum and leaving a small akinetic part. It can be argued that, in the same way a small myocardial infarction is less likely to lead to negative remodeling [18,19], the smaller the patch (i.e. the smaller the residual akinetic region), the greater the probability of positive remodeling. Incidentally, we observed a completely silent delay enhancement sequence in some patients who performed magnetic resonance at later follow-up visit except for the small area of the patch embedded in the functioning myocardium (Figure 5).

In this way, volume can be reduced by excluding the anteroseptal region and giving the apex a triangular shape [20-22]. A narrow patch reduces the distance from the contractile myocardium while simultaneously absorbing the stress of the suture. It must be pointed out that we refer to the true and not the sutured dimensions of the patch, which are even smaller: reducing the long and short dimensions by 0.4 mm (all around the suture line), the mean patch area is 2.5 ± 1.2 cm^2^. Finally, the patch should only act as a support for the suture line (unloading the stress) and guide the rebuilding of the apex in order to obtain a ventricular elliptic shape.

A small patch as in our series can also help rebuild near-normal ventricular volumes because it does not add any volume itself. Reported data in large series [23] show that postoperative ESVI is usually higher than 45 mL/m^2 ^(ESVI is 70 ± 6.3 mL/m^2 ^in akinetic and 48 ± 2.9 mL/m^2 ^in dyskinetic ventricles) and the same limitation affects the use of a large oval patch [24] (60 × 30 mm, area = 14.1 cm^2^, postoperative ESVI = 49 ± 18 mL/m^2^) which could also be the basis for later redilation.

## Conclusion

This study demonstrates that:

a) a surgical method which equalizes the importance of volume and shape and leaves a small akinetic area leads to a ventricular restoration procedure as physiologic as possible to obtain steady geometrical and functional outcome;

b) wall motion score index and percentage of akinesia are key parameters to monitor positive remodeling;

c) a complete set of data is important when assessing left ventricular performance at serial follow-up visits;

d) a wider indication to repair mitral regurgitation is also required because even mild regurgitation is not surely eliminated by surgical restoration [25].

In conclusion, restoration surgery should aim at rebuilding a nearly normally shaped left ventricle with a near-normal volume, which guarantees better myocyte and chamber physiology [26], and leaving a smaller area of residual akinesia, all of which lead to less negative remodeling over time. Optimisation of the anterior restoration technique can achieve lasting results and allow patients to benefit from long-term gains in terms of functional class and the quality of life.

## Study limitations

Although the number of patients is small, this is compensated for by their strict selection, the completeness of the follow-up examinations and the highly significant *P *values: it is quite rare to obtain such homogeneous results in a small series.

A longer follow-up is necessary to confirm the slow progression rate reported in our series and define the extent to which the described technique slows or prevents redilation, and for how long. Critical analysis of the data shows that there is still a slight tendency toward redilation, but it is only about 1 mL per year (ESVI, FU 3 – FU 1, mean value). If this estimate of progression remains the same over time, it will take more than 40 years before the "new ventricles" return to their preoperative status, save for new negative events.

This study lacks a control group. Literature dealing with ischemic cardiomyopathy comprehends many non-randomized studies reporting outcomes in patients treated with or without ventricular reconstruction and with or without endoventricular patch, using different indexes of left ventricular performance [3-11,20,23,24]. The ongoing Surgical Treatment for Ischemic Heart Failure trial will address open issues about surgical correction of ischemic left ventricular dysfunction and which will be the role of surgical ventricular restoration in this disease. We entered the trial in June 2003 and this was the occasion for reviewing this selected series of patients treated by surgical anterior ventricular restoration with a serial, complete, echocardiographic, long-term follow-up assessment to have the highest level of information available. Our aim was just to highlight some technical, echocardiographic, functional and follow-up issues of this kind of surgical treatment.

## Competing interests

The author(s) declare that they have no competing interests.

## Authors' contributions

MC cured the conception and design of the study, performed surgical operations and conceived analysis of data. AA carried out all of the echocardiographic studies. EV, MDT, ZM assisted in operative and postoperative care. MC, FB, GT, EQ cured the drafting and revising of the manuscript. All authors read and approved the final manuscript.
